# Hard Tick Surveillance in the Republic of Korea, 2023–2025

**DOI:** 10.56786/PHWR.2026.19.42.1

**Published:** 2026-11-05

**Authors:** Hyeon Seung Lee, Jinsung Seo, Jung-Won Ju, Hee-Il Lee

**Affiliations:** Division of Vectors and Parasitic Diseases, Department of Disease Diagnosis and analysis, Korea Disease Control and Prevention Agency Cheongju Korea

**Keywords:** Ticks, Surveillance, Seasonal occurrence, Distribution

## Abstract

**Objectives:**

To characterize annual and seasonal tick occurrence and species distribution using surveillance data from 16 locations in the Republic of Korea (ROK) during 2023–2025 and to descriptively compare monthly mean temperature with tick abundance.

**Methods:**

Tick surveillance was conducted monthly from April to November. Ticks were collected using carbon dioxide-baited traps along mountain trails and in graves, copses, and grasslands. Adults and nymphs were morphologically identified to species level, while larvae were identified to genus level. Tick density was expressed as the mean number of ticks collected per trap (tick index), and monthly changes in temperature and the tick index were descriptively compared.

**Results:**

A total of 161,318 ticks were collected. Tick abundance increased in May, decreased during June–July, and peaked during August–September. Nymphs, adults, and larvae were most abundant in May, July, and August–September, respectively, with larvae comprising the largest proportion of the total collection and predominating in August–September. *Haemaphysalis longicornis* was the predominant species, accounting for >95% of species-level identifications. Ticks were widely distributed, with relatively high densities in Gangwon State and Jeju Special Self-Governing Province.

**Conclusions:**

Nationwide surveillance revealed distinct seasonal patterns among developmental stages, with peak tick abundance in August–September. Consistent with previous surveillance, *H. longicornis* was the predominant species. These findings provide baseline data on tick occurrence in the ROK and support the development of strategies for tick-borne disease surveillance and prevention.

**Key messages**

**① What is known previously?**

Ticks are major vectors of severe fever with thrombocytopenia syndrome and other tick-borne diseases, with distinct seasonal patterns among developmental stages.

**② What new information is presented?**

A comparison across 16 locations during 2023–2025 showed that the tick index and nymph abundance were highest in 2024. Across all 3 years, nymphs, adults, and larvae were most abundant in May, July, and August–September, respectively, with *Haemaphysalis longicornis* being the predominant species.

**③ What are implications?**

Long-term nationwide surveillance data can enhance understanding of tick population dynamics and inform tick-borne disease prevention and control.

## Introduction

Hard ticks are representative hematophagous arthropod vectors that parasitize humans and animals and transmit various pathogens. A total of five genera and 44 species of ticks have been reported in the Republic of Korea (ROK). Among them, *Haemaphysalis longicornis* is known to be the dominant species in the ROK and is recognized as a major vector of severe fever with thrombocytopenia syndrome (SFTS) [1]. In addition, SFTS virus (SFTSV) has been detected in other hard tick species, including *Haemaphysalis flava*, *Amblyomma testudinarium*, and the *Ixodes nipponensis* [2].

SFTS is a representative tick-borne zoonotic infectious disease caused by SFTSV, and after it was first reported in China in 2009, cases have continuously occurred mainly in the ROK, Japan, and China [3]. In the ROK, SFTS cases have been reported every year since the first case in 2013, and it is recognized as an important public health issue due to its high case fatality rate [4]. Although most are known to be infected through the bite of virus-carrying hard ticks, rare cases of infection through contact with the body fluids or blood of infected patients or companion animals have also been reported [5,6].

Hard ticks are known as three-host ticks that feed on different hosts across three developmental stages—larvae, nymphs, and adults—and exhibit different seasonal occurrence patterns by developmental stage [7]. In addition, the occurrence density and activity period of hard ticks are known to be influenced by various meteorological and environmental factors, such as temperature, precipitation, humidity, and vegetation environment [8]. Recently, the possibility that the habitat range and activity period of hard ticks will expand due to the rise in mean temperature and ecological changes resulting from climate change has been raised, and consequently, there are concerns that the risk of occurrence of tick-borne infectious diseases will also increase [9].

The Korea Disease Control and Prevention Agency has been continuously conducting nationwide surveillance to understand the occurrence status of domestic hard ticks. However, studies comparing and analyzing the association between the annual and seasonal occurrence patterns of domestic hard ticks and meteorological factors over a long period are limited. Therefore, based on the results of hard tick surveillance conducted in 16 regions nationwide from 2023 to 2025, this study analyzed the seasonal occurrence characteristics of each year, identified the distribution of major hard tick species, and compared monthly mean temperatures with hard tick occurrence patterns to provide baseline data for establishing future tick-borne infectious disease prevention and control policies.

## Methods

### 1. Study Sites and Environments

To understand nationwide hard tick occurrence patterns, surveillance was conducted once in the third week of each month from April to November annually from 2023 to 2025. The survey was conducted jointly with 16 regional climate change vector surveillance hub centers nationwide. The total number of survey areas by year was 32 in 2023, 16 in 2024, and 20 in 2025. To compare the annual occurrence patterns while minimizing the impact of differences in the number of survey areas, data from 16 areas where surveys were conducted in all three years were analyzed. The analyzed areas included Gangwon State (Inje-gun and Samcheok-si), Incheon Metropolitan City (Ganghwa-gun), Ulsan Metropolitan City (Ulju-gun), Gyeonggi-do (Pocheon-si and Gwangju-si), Chungcheongbuk-do (Chungju-si), Chungcheongnam-do (Nonsan-si and Dangjin-si), Jeonbuk State (Gochang-gun), Jeollanam-do (Gokseong-gun and Boseong-gun), Gyeongsangbuk-do (Gimcheon-si and Andong-si), Gyeongsangnam-do (Jinju-si), and Jeju Special Self-Governing Province (Jeju-si). In each survey area, four environments with a high probability of contact between humans and hard ticks were surveyed: mountain trails, graves, copses, and grasslands.

### 2. Hard Tick Collection and Classification

On each survey day, three traps utilizing solid carbon dioxide (dry ice) as an attractant were installed for each environment, operating a total of 12 traps per region. The traps were installed at 10:00 AM on the survey day and operated for a total of 24 hours until 10:00 AM the following day, after which specimens were recovered. The collected hard ticks were classified and identified using a dissecting microscope and morphological keys [10]. The larvae, due to limitations in morphological identification, were classified only to the genus level, whereas adults and nymphs were identified to the species level. Additional molecular identification was not performed.

### 3. Climate Data and Spatial Analysis

Hard tick density was calculated based on the mean number of individuals per trap (∑number of individuals/∑number of traps) and was expressed as the hard tick index. The number of hard tick individuals was measured by including all adults, nymphs, and larvae. Climate data utilized the synoptic meteorological observation data from the Weather Data Open Portal of the Korea Meteorological Administration, and data from the meteorological observation station adjacent to each survey site were applied [11]. Yearly variation patterns of the monthly mean temperature and the hard tick index were descriptively compared, and the mean temperature during the overwintering period was calculated by arithmetically averaging the monthly mean temperatures from December of the previous year to February of the current year. Statistical association between meteorological factors and hard tick occurrence was not analyzed. The spatial distribution of hard ticks was visualized using the Inverse Distance Weighted technique of ArcGIS (Environmental Systems Research Institute).

## Results

During the hard tick surveillance conducted from April to November of 2023–2025, a total of 161,318 individual hard ticks were collected (Table 1). By year, the number of collected individuals was 45,071 in 2023, 71,551 in 2024, and 44,696 in 2025. The monthly tick index increased in May, decreased in June–July, peaked in August–September, and then declined. By developmental stage, larvae accounted for the highest proportion, with 21,865 individuals collected in 2023, 34,774 in 2024, and 26,292 in 2025. Nymphs showed a relatively high density in spring (May), and adults showed a high density in summer (July). For larvae, the density began to gradually increase from July, showing the highest density in August–September. In addition, larvae accounted for more than 80% of the total collected individuals in August and September (Figure 1). The yearly variation patterns of the monthly mean temperature and the tick index were compared and presented (Figure 2). The mean temperature during the overwintering period (December of the previous year to February of the current year) was 0.1℃ in 2023, 2.5℃ in 2024, and 0.2℃ in 2025, being the highest in 2024. The tick index in 2023 was the highest in September (56.6), and in 2024 and 2025, it showed the highest values in August at 89.8 and 63.5, respectively. The mean temperature was also the highest in July–August, and subsequently, the tick index tended to decrease along with the decrease in temperature.

Regarding collection results by habitat, 20,260, 29,118, and 14,595 individuals were collected from grasslands in 2023, 2024, and 2025, respectively, accounting for the highest proportion of the total number of individuals collected (Table 2). By species, *H. longicornis* was identified as the dominant species in all survey years. For *H. longicornis*, the numbers of adults and nymphs, excluding larvae, were 22,089 in 2023, 35,757 in 2024, and 17,557 in 2025, accounting for approximately 96.2% of the cumulative three-year total. In addition, *H. flava* (3.5%), *A. testudinarium* (0.2%), *I. nipponensis* (0.1%), *Haemaphysalis japonica*, and *Haemaphysalis formosensis* were identified. The nationwide distribution survey confirmed that hard ticks were widely distributed across the surveyed areas (Figure 3). In particular, tick density was found to be high in Gangwon State and Jeju Special Self-Governing Province.

## Discussion

Surveillance of hard ticks conducted in 16 regions nationwide from 2023 to 2025 confirmed their nationwide distribution, with occurrence varying by year. The annual mean tick index was 29.3 in 2023, 46.6 in 2024, and 29.1 in 2025, with the highest tick density recorded in 2024. In particular, a total of 71,551 individuals were collected in 2024, which was markedly higher than the numbers collected in 2023 (45,071 individuals) and 2025 (44,696 individuals). By developmental stage, 27,400 nymphs were collected in 2024, representing the largest proportion across the years, being 89.2% and 111.7% more than the nymphs collected in 2023 (14,482 individuals) and 2025 (12,945 individuals), respectively. In addition, 8,860 nymphs were collected in May 2024, the highest number recorded during the survey period. Comparison of meteorological data showed that the mean temperature in April 2024 was 15.1℃, higher than in 2023 (13.2℃) and 2025 (13.1℃). The mean temperature in May 2024 was 17.7℃, higher than in 2025 (16.8℃) and similar to that in 2023 (18.1℃). Hard ticks are known to overwinter mainly in the nymphal stage and begin activity in spring, and the scale of nymph occurrence is known to affect the subsequent formation of adult and larval populations [7]. Nymphs emerging in spring are individuals that developed from larvae in the previous year and overwintered. Comparing mean temperatures during the overwintering periods (December–February) preceding the spring nymph population in each year, the mean temperature from December 2023 to February 2024, corresponding to the overwintering period of the 2024 nymphs, was 2.5℃. This temperature was more than 2℃ higher than the mean temperatures during the overwintering periods of the 2023 nymphs (December 2022–February 2023, 0.1℃) and the 2025 nymphs (December 2024–February 2025, 0.2℃). These relatively mild overwintering temperatures may have partially influenced the overwintering survival and spring activity of the 2024 nymphs. However, the increase in the number of nymphs in 2024 was presumed to have been influenced not only by overwintering temperatures but also by carry-over effects, including the scale of larval occurrence in the previous year, climatic and environmental conditions, and overwintering survival rates during winter. In this survey, both nymph and larval numbers were also the highest in 2024, which showed the highest tick density, suggesting that the scale of spring nymph occurrence may be associated with the increase in overall hard tick density in that year. On the other hand, although larval occurrence was the highest in 2024, the number of collected nymphs and overall number of hard ticks in 2025 decreased compared with 2024. This finding suggests that the number of nymphs collected in the following year is not determined solely by the scale of larval occurrence in the previous year, and that environmental conditions, such as the relatively lower overwintering and spring temperatures in 2025, may have partially influenced the survival and activity of overwintering nymphs. However, because larval blood-feeding, molting, overwintering survival rates, and other environmental factors were not directly investigated or statistically analyzed, it is difficult to conclude that the decrease in 2025 was attributable to any specific factor. Therefore, annual variations in hard tick occurrence should be interpreted cautiously, taking into account not only the temperature of the corresponding year but also the population size of the previous year, environmental conditions during the overwintering period, precipitation, humidity, host animal density, and habitat environment [8,12]. Long-term studies are needed to clarify the relationship between these factors and variations in hard tick populations.

Looking at the monthly occurrence patterns during the survey period, the tick index showed a common tendency to increase in April–May, decrease in June–July, and then rapidly increase again in August–September. In particular, the tick index tended to increase during periods of rising mean temperatures, and the number of individuals increased significantly in summer when the mean temperature remained above 20℃. On the other hand, despite the period of high mean temperatures, the tick index temporarily decreased in June–July, which is estimated to be due to environmental changes within the habitat resulting from increased precipitation during the rainy season affecting the activity of hard ticks. By developmental stage, clear seasonal differences were confirmed, with nymphs showing the highest density in May, adults in July, and larvae in August–September. Hard ticks mainly overwinter in the nymphal stage, so an increase in the number of nymphs is mostly observed in spring. They are known to grow into adults and lay eggs throughout June and July, and the density of the population increases significantly as larvae hatch from the eggs laid from August to September [7]. In particular, the seasonal increase in the domestic hard tick population is considered to be driven by larval occurrence.

In the collection results by environment, the grassland environment showed the highest density in all years, with 20,260, 29,118, and 14,595 individuals collected in 2023, 2024, and 2025, respectively, accounting for the highest proportion of the total collected individuals. This finding might be attributed to the grassland environment, which provides suitable conditions for hard tick habitation and host animal activity, which was consistent with previous surveillance results [13,14]. By species, five species across three genera were confirmed in 2023, and six species across three genera were confirmed in 2024–2025. *H. longicornis* and *H. flava* were distributed nationwide, while *A. testudinarium* was identified mainly in the southern regions, such as Jinju-si and Ulsan Metropolitan City. The number of collected *I. nipponensis* was very small except in some regions, and *H. japonica, H. formosensis*, and *I. persulcatus* were mainly identified in specific areas, such as mountainous areas or southern regions [13,14]. In particular, *H. longicornis*, known as the major vector species of SFTS, was again confirmed to be the dominant species in the ROK, and *H. flava*, *A. testudinarium*, and *I. nipponensis*, for which SFTSV detection has been reported, were also confirmed in all survey years.

These seasonal and environmental occurrence patterns were similar to previous domestic surveillance results. In the 2022–2024 surveillance data, nymphs showed high density mainly in spring, adults in summer, and larvae in August–September, and the pattern of many individuals being collected in grasslands and *H. longicornis* dominating was repeatedly confirmed [13-15]. This finding shows that the seasonality of domestic hard ticks by developmental stage, key habitat environments, and the characteristics of the dominant species are generally maintained year by year. However, since there are differences in the survey regions and aggregation scope between existing annual report data and the present study, caution is required when directly comparing numerical values of the total collected individuals and the tick index.

SFTS is a representative tick-borne infectious disease with patients continuously occurring every year in the ROK. In previous domestic surveillance data, it was reported that with the marked increase in the density of hard tick larvae in August–September, the occurrence of SFTS patients increased in autumn, peaking in October [13-15]. Consistently, in the present study, the density of hard tick larvae increased significantly in August–September, showing an occurrence pattern similar to that reported in previous surveillance data. This period overlaps with the time when outdoor activities, such as visiting ancestral graves and farming operations, increase, which may increase the possibility of contact between humans and hard ticks. SFTSV has been reported to be capable of transovarial transmission in *H. longicornis* [1,16], so larvae can also potentially harbor SFTSV. Recently, the possibility of expanding the habitat range and activity period of hard ticks has been raised due to rising mean temperatures and ecological and environmental changes caused by climate change, and accordingly, the risk of tick-borne infectious diseases may also increase [8,9,12]. This study confirmed the annual and seasonal occurrence characteristics and species distribution of domestic hard ticks through 3-year surveillance data collected from 16 regions nationwide, and suggested the possibility that the scale of spring nymph occurrence may be related to the overall hard tick density of the corresponding year.

Since this study analyzed once-monthly surveillance data from 16 regions commonly included across the three years for inter-annual comparison, it may not have fully reflected the spatial variation of all survey regions by year and short-term changes in hard tick density. In addition, meteorological and environmental factors were descriptively compared with hard tick occurrence patterns, and linked analysis with pathogen detection results and patient occurrence data was not performed, limiting the evaluation of a direct relationship between changes in hard tick density and infection risk.

Despite these limitations, this study is significant in that it confirmed the annual and seasonal occurrence characteristics and species distribution of domestic hard ticks based on surveillance data conducted in the same 16 regions for three years. The results of this study can be used as basic data to understand domestic hard tick occurrence patterns and to establish tick-borne infectious disease surveillance and prevention strategies. Continuous surveillance, along with long-term ecological studies linking climate factors, host animal density, pathogen detection results, and patient occurrence data, are required in the future.

## Figures and Tables

**Figure 1:**
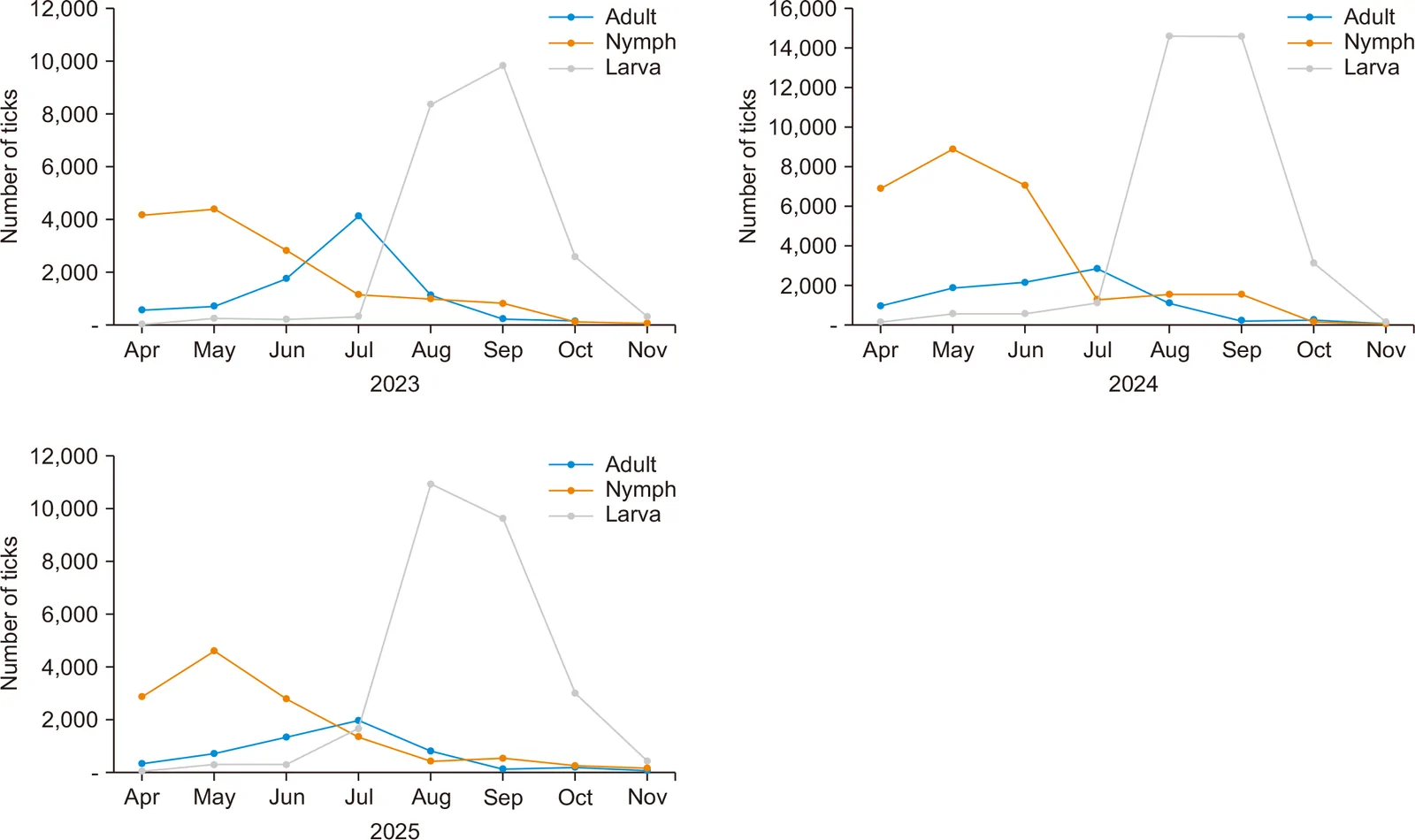
Monthly occurrence patterns of ticks by developmental stage in the Republic of Korea, 2023–2025

**Figure 2:**
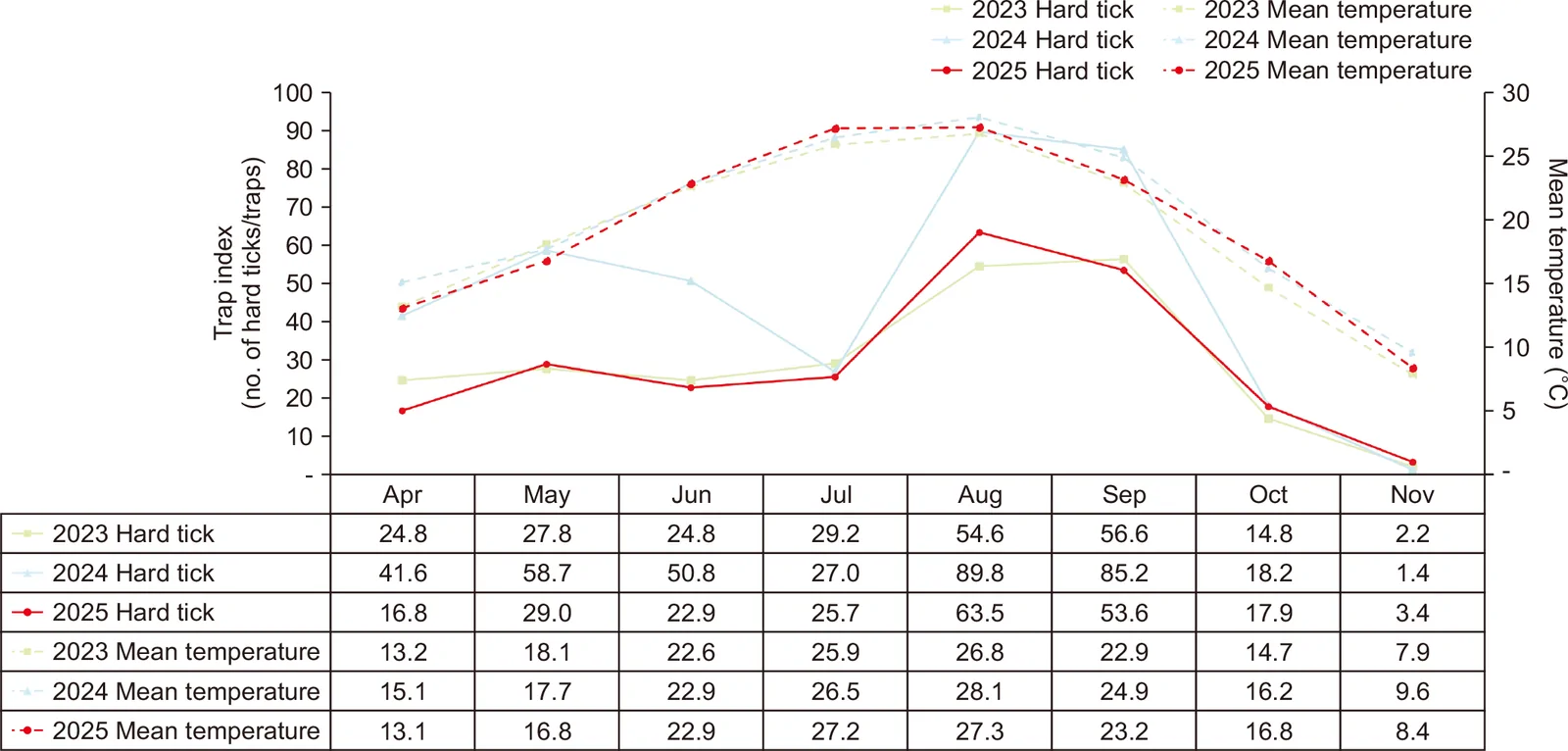
Monthly tick index and mean temperature changes in the Republic of Korea, 2023–2025

**Figure 3:**
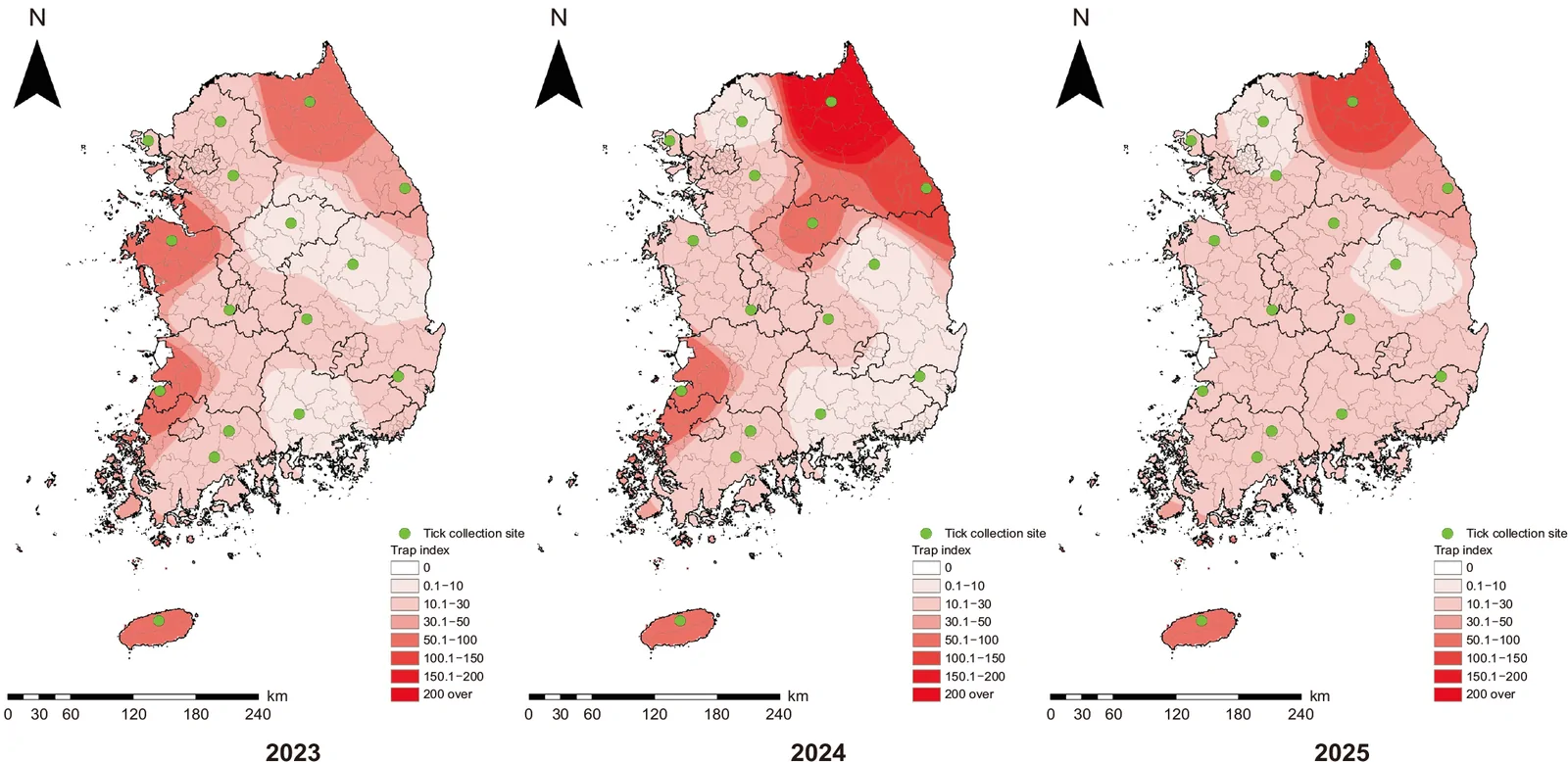
Spatial distribution of tick occurrence in the Republic of Korea, 2023–2025

**Table 1:** Number of ticks collected by developmental stage during the surveillance period (April–November) in 2023–2025

Year	Developmental stage	Month								Total
		Apr	May	Jun	Jul	Aug	Sep	Oct	Nov	
2023	Adult	555	706	1,746	4,139	1,122	238	147	71	8,724
	Nymph	4,171	4,384	2,815	1,157	988	788	118	61	14,482
	Larvae	33	241	202	307	8,369	9,834	2,584	295	21,865
	Subtotal	4,759	5,331	4,763	5,603	10,479	10,860	2,849	427	45,071
2024	Adult	956	1,856	2,141	2,825	1,108	204	240	47	9,377
	Nymph	6,897	8,860	7,059	1,269	1,535	1,565	150	65	27,400
	Larvae	137	558	546	1,088	14,594	14,583	3,110	158	34,774
	Subtotal	7,990	11,274	9,746	5,182	17,237	16,352	3,500	270	71,551
2025	Adult	320	692	1,315	1,958	809	117	175	73	5,459
	Nymph	2,869	4,602	2,794	1,345	421	519	245	150	12,945
	Larvae	29	276	282	1,635	10,967	9,659	3,013	431	26,292
	Subtotal	3,218	5,570	4,391	4,938	12,197	10,295	3,433	654	44,696

**Table 2:** Number of ticks collected by species and collection environment during 2023–2025

Year	Species	Environment				Total
		Grassland	Mountain trail	Copse	Grave	
2023	*Haemaphysalis longicornis*	10,169	3,090	4,374	4,456	22,089
	*Haemaphysalis flava*	344	276	172	212	1,004
	*Amblyomma testudinarium*	24	14	26	4	68
	*Ixodes nipponensis*	15	5	5	11	36
	*Haemaphysalis japonica*		1	5	3	9
	Larvae (*Haemaphysalis* spp.)	9,708	3,917	2,105	6,134	21,864
	Larvae (*Ixodes* spp.)		1			1
	Subtotal	20,260	7,304	6,687	10,820	45,071
2024	*H. longicornis*	18,745	4,446	7,470	5,096	35,757
	*H. flava*	183	264	167	308	922
	*A. testudinarium*	22	11	15	13	61
	*I. nipponensis*	10	5	4	6	25
	*H. japonica*	3	4	1	2	10
	*Haemaphysalis formosensis*				2	2
	Larvae (*Haemaphysalis* spp.)	10,155	6,059	5,969	12,590	34,773
	Larvae (*Amblyomma* spp.)			1		1
	Subtotal	29,118	10,789	13,627	18,017	71,551
2025	*H. longicornis*	7,721	3,052	3,517	3,267	17,557
	*H. flava*	198	271	141	175	785
	*A. testudinarium*	3	2	6	3	14
	*I. nipponensis*	10	3	5	22	40
	*H. japonica*	5		1		6
	*Ixodes persulcatus*	2				2
	Larvae (*Haemaphysalis* spp.)	6,656	4,663	6,424	8,549	26,292
	Subtotal	14,595	7,991	10,094	12,016	44,696
