# Supplementary material for: Hard Tick Surveillance in the Republic of Korea, 2023–2025
Source: Public Health Wkly Rep. 2026 Nov 5;19(42):1737–55. doi: 10.56786/PHWR.2026.19.42.1 (PMC13740383; doi:10.56786/PHWR.2026.19.42.1)
Supplement: Translation

# Translation: 2023-2025년 국내 참진드기 감시 현황

**Authors:** 현승 이, 진성 서, 정원 주, 희일 이

**Affiliations:** 질병관리청 진단분석국 매개체분석과

**Keywords:** 참진드기, 감시, 계절 발생 양상, 분포

## Abstract

**목적:**

이번 연구는 2023–2025년 전국 16개 지역에서 수행한 참진드기 감시 결과를 바탕으로 국내 참진드기의 연도별ㆍ계절별 발생 양상과 종 분포 특성을 분석하고 월별 평균기온과 참진드기 발생 양상을 비교하고자 하였다.

**방법:**

2023년부터 2025년까지 매년 4월부터 11월까지 월 1회 참진드기 감시를 수행하였다. 산길, 무덤, 잡목림 및 초지 등 다양한 환경에서 이산화탄소 유인 채집기를 이용하여 참진드기를 채집하였으며 성충과 약충은 종 수준, 유충은 속 수준으로 형태학적으로 동정하였다. 참진드기 밀도는 채집기당 포집된 평균 개체수를 참진드기 지수로 산출하였고 월별 평균기온과 참진드기 지수의 변화 양상을 기술적으로 비교하였다.

**결과:**

2023–2025년 동안 총 161,318개체의 참진드기가 채집되었다. 참진드기 발생은 5월에 증가한 후 6–7월 일시적으로 감소하였으며 8–9월에 가장 높은 밀도를 나타냈다. 성장단계별로는 약충이 5월, 성충이 7월, 유충이 8–9월에 가장 높은 발생을 보였다. 유충은 전체 채집 개체 중 가장 높은 비율을 차지하였으며, 특히 8–9월 채집 개체의 대부분을 차지하였다. 종별로는 작은소피참진드기(*Haemaphysalis longicornis*)가 우점종으로 확인되었으며 유충을 제외한 개체의 95% 이상을 차지하였다. 참진드기는 전국적으로 분포하였으며 강원특별자치도와 제주특별자치도에서 상대적으로 높은 밀도를 나타냈다.

**결론:**

2023–2025년 전국 감시 결과, 국내 참진드기는 전국적으로 분포하였으며 8–9월에 가장 높은 발생 밀도를 나타냈다. 또한 작은소피참진드기가 기 보고된 바와 같이 우점종으로 확인되었고, 성장단계에 따라 뚜렷한 계절적 발생 양상을 보였다. 이번 연구 결과는 국내 참진드기의 연도별ㆍ계절별 발생 특성을 이해하고 향후 진드기 매개감염병 감시 및 예방 전략 수립을 위한 기초자료로 활용될 수 있을 것으로 판단된다.

**핵심요약**

**① 이전에 알려진 내용은?**

참진드기는 중증열성혈소판감소증후군 등의 주요 매개체로 알려져 있으며 성장단계에 따라 뚜렷한 계절적 발생 양상을 나타낸다.

**② 새로이 알게 된 내용은?**

2023–2025년 전국 16개 지역의 참진드기 발생 양상을 비교한 결과 2024년에 연평균 참진드기 지수와 약충 채집 개체수가 모두 가장 높았다. 3개 연도 모두 약충은 5월, 성충은 7월, 유충은 8–9월에 가장 높은 밀도를 보였으며 작은소피참진드기가 우점종으로 확인되었다.

**③ 시사점은?**

전국 단위의 장기 감시자료는 국내 참진드기의 계절적ㆍ연도별 발생 특성을 파악하고 진드기 매개감염병 예방ㆍ관리 전략을 수립하기 위한 기초자료로 활용될 수 있다.

## 서 론

참진드기는 사람과 동물에 기생하여 다양한 병원체를 전파하는 대표적인 흡혈성 절지동물 매개체로 국내에서는 총 5속 44종이 보고되어 있다. 이 중 작은소피참진드기(*Haemaphysalis longicornis*)는 국내에서 가장 우점하는 종으로 알려져 있으며, 특히 중증열성혈소판감소증후군(severe fever with thrombocytopenia syndrome, SFTS)의 주요 매개종으로 알려져 있다[1]. 이외에도 개피참진드기(*Haemaphysalis flava*), 뭉뚝참진드기(*Amblyomma testudinarium*), 일본참진드기(*Ixodes nipponensis*) 등 참진드기 종에서 SFTS virus (SFTSV)가 검출된 바 있다[2].

SFTS는 SFTSV에 의해 발생하는 대표적인 진드기 매개 인수공통감염병으로 2009년 중국에서 처음 보고된 이후 한국, 일본, 중국을 중심으로 지속적으로 환자가 발생하고 있다[3]. 국내에서는 2013년 첫 환자 발생 이후 매년 환자가 보고되고 있으며 높은 치명률로 인해 중요한 공중보건학적 문제로 인식되고 있다[4]. 대부분 바이러스를 보유한 참진드기에 물려 감염되는 것으로 알려져 있으나, 드물게 감염 환자나 반려동물의 체액, 혈액 등과 접촉을 통하여 감염된 사례도 보고되고 있다[5,6].

참진드기는 유충, 약충 및 성충의 세 단계에 걸쳐 서로 다른 숙주를 흡혈하는 3숙주 진드기로 알려져 있으며 성장단계별로 서로 다른 계절적 발생 양상을 보인다[7]. 또한 참진드기의 발생 밀도와 활동 시기는 기온, 강수량, 습도 및 식생 환경과 같은 다양한 기상ㆍ환경요인의 영향을 받는 것으로 알려져 있다[8]. 최근 기후변화에 따른 평균기온 상승과 생태환경 변화로 참진드기의 서식 범위와 활동 기간이 확대될 가능성이 제기되고 있으며 이에 따라 진드기 매개감염병의 발생 위험 또한 증가할 것으로 우려되고 있다[9].

질병관리청에서는 국내 참진드기 발생 현황을 파악하기 위한 전국 단위 감시를 지속적으로 수행하고 있다. 그러나 국내 참진드기의 연도별 및 계절별 발생 양상과 기상요인 간의 연관성을 장기간 비교ㆍ분석한 연구는 제한적인 실정이다. 따라서 이번 연구에서는 2023년부터 2025년까지 전국 16개 지역에서 수행한 참진드기 감시 결과를 바탕으로 각 연도의 계절별 발생 특성을 분석하고, 주요 참진드기 종의 분포를 확인하며 월별 평균기온과 참진드기 발생 양상을 비교하여 향후 진드기 매개 감염병 예방 및 관리 정책 수립을 위한 기초자료를 제공하고자 하였다.

## 방 법

### 1. 조사 지점 및 환경

전국 참진드기 발생 양상을 파악하기 위해 2023년부터 2025년까지 매년 4월부터 11월까지 매월 셋째 주에 1회씩 수행되었다. 조사는 전국 16개 권역별 기후변화 매개체 감시 거점센터와 공동으로 수행하였다. 연도별 전체 조사 지역은 2023년 32개, 2024년 16개, 2025년 20개였으며 조사 지역 수의 차이에 따른 영향을 최소화하여 연도별 발생 양상을 비교하기 위해 3개 연도 모두에서 조사가 수행된 16개 지역의 자료를 분석하였다. 분석 대상 지역은 강원특별자치도(인제군ㆍ삼척시), 인천광역시(강화군), 울산광역시(울주군), 경기도(포천시ㆍ광주시), 충청북도(충주시), 충청남도(논산시ㆍ당진시), 전북특별자치도(고창군), 전라남도(곡성군ㆍ보성군), 경상북도(김천시ㆍ안동시), 경상남도(진주시) 및 제주특별자치도(제주시)였다. 각 조사 지역에서는 사람과 참진드기의 접촉 가능성이 높은 산길, 무덤, 잡목림 및 초지의 4개 환경을 조사하였다.

### 2. 참진드기 채집 및 분류

각 조사일에 고체 이산화탄소(드라이아이스)를 유인물질로 활용한 채집기를 환경별 3개씩 설치하여 지역별로 총 12개를 운영하였다. 채집기는 조사일 오전 10시에 설치하고 다음 날 오전 10시까지 총 24시간 동안 운영하고 검체를 회수하였다. 수거된 참진드기는 해부현미경과 형태학적 검색표를 이용하여 분류ㆍ동정하였다[10]. 유충의 경우 형태학적 동정에 제한이 있어 속(genus) 수준까지만 구분하였으며 성충과 약충은 종(species) 수준까지 동정하였다. 추가적인 분자생물학적 동정은 수행하지 않았다.

### 3. 기후자료 및 공간분석

참진드기 밀도는 채집기당 평균 개체수(∑개체수/∑채집기 수)를 기반으로 계산하였으며 참진드기 지수로 표현하였다. 참진드기 개체수는 성충, 약충, 유충을 모두 포함하여 측정하였다. 기후자료는 기상청 기상자료 개방포털의 종관기상관측자료를 활용하였으며 각 조사 지점에 인접한 기상관측소의 자료를 적용하였다[11]. 월별 평균기온과 참진드기 지수의 연도별 변화 양상을 기술적으로 비교하였으며 월동기 평균기온은 전년도 12월부터 당해 연도 2월까지의 월평균기온을 산술평균하여 산출하였다. 기상요인과 참진드기 발생 간의 통계적 연관성 분석은 수행하지 않았다. 참진드기 공간 분포는 ArcGIS (Environmental Systems Research Institute)의 Inverse Distance Weighted 기법을 이용하여 시각화하였다.

## 결 과

2023–2025년 4월부터 11월까지 수행된 참진드기 감시를 통해 총 161,318개체의 참진드기가 채집되었다(표 1). 연도별 채집 개체수는 2023년 45,071개체, 2024년 71,551개체, 2025년 44,696개체였다. 월별 발생 양상은 5월에 일시적으로 증가한 이후 6–7월에는 감소한 뒤 8–9월에 높은 밀도를 보인 후 감소하는 경향을 나타냈다. 발생단계별로는 유충이 가장 높은 비율을 차지하였으며 2023년 21,865개체, 2024년 34,774개체, 2025년 26,292개체가 채집되었다. 약충은 봄철(5월)에 상대적으로 높은 밀도를 보였으며 성충은 여름철(7월)에 높은 밀도를 보였다. 유충은 7월부터 밀도가 서서히 증가하기 시작하여 8–9월에 가장 높은 밀도를 보였으며 8월과 9월의 전체 채집 개체수의 약 80% 이상은 유충이었다(그림 1). 월별 평균기온과 참진드기 지수의 연도별 변화 양상을 비교하여 제시하였다(그림 2). 월동기(전년도 12월–당해 연도 2월) 평균기온은 2023년 0.1℃, 2024년 2.5℃, 2025년 0.2℃로 2024년에 가장 높았다. 2023년 참진드기 지수는 9월(56.6)에 가장 높았으며 2024년과 2025년은 각각 8월에 89.8 및 63.5로 가장 높은 값을 보였다. 평균기온 또한 7–8월에 가장 높게 나타났으며 이후 기온 감소와 함께 참진드기 지수도 감소하는 양상을 보였다.

환경별 채집 결과에서는 초지에서 2023년 20,260개체, 2024년 29,118개체, 2025년 14,595개체가 채집되어 전체 채집 개체수 중 가장 높은 비율을 차지하였다(표 2). 종별로는 작은소피참진드기가 모든 조사 연도에서 우점종으로 확인되었다. 작은소피참진드기는 유충을 제외한 성충과 약충 개체수가 2023년 22,089개체, 2024년 35,757개체, 2025년 17,557개체로 3년 합산 기준 약 96.2%를 차지하였다. 이외에도 개피참진드기(3.5%), 뭉뚝참진드기(0.2%), 일본참진드기(0.1%), 사슴피참진드기 및 산림참진드기 등이 확인되었다. 전국 단위 분포 조사 결과 참진드기는 조사 지역 전반에 걸쳐 광범위하게 분포하는 것으로 확인되었다(그림 3). 특히 강원특별자치도 지역과 제주특별자치도 지역에서 밀도가 높은 것으로 확인되었다.

## 논 의

2023–2025년 전국 16개 지역에서 수행한 참진드기 감시 결과, 참진드기는 전국적으로 분포하는 것으로 확인되었으며 연도별 발생의 차이를 나타내었다. 연도별 평균 참진드기 지수는 2023년 29.3, 2024년 46.6, 2025년 29.1로 확인되었으며, 2024년이 가장 높은 발생 밀도를 나타냈다. 특히 2024년에는 총 71,551개체가 채집되어 2023년(45,071개체) 및 2025년(44,696개체)보다 현저히 많은 개체가 확인되었다. 발생단계별로 살펴보면 2024년 약충 채집 개체수는 27,400개체로 2023년(14,482개체) 대비 약 89.2%, 2025년(12,945개체) 대비 약 111.7% 증가하여 가장 큰 차이를 나타냈다. 또한 2024년 5월 약충 채집 개체수는 8,860개체로 조사기간 중 가장 높은 수준을 보였다. 기상자료를 비교한 결과, 2024년 4월 평균기온은 15.1℃로 2023년(13.2℃)과 2025년(13.1℃)보다 높게 나타났으며 5월 평균기온은 17.7℃로 2025년(16.8℃)보다는 높고 2023년(18.1℃)과 유사한 수준이었다. 참진드기는 주로 약충 단계로 월동한 후 봄철 활동을 시작하며, 약충의 발생 규모는 이후 성충 및 유충 개체군 형성에 영향을 미치는 것으로 알려져 있다[7]. 봄철에 출현하는 약충은 전년도 유충에서 발육하여 월동한 개체군이다. 각 연도의 봄철 약충 발생에 선행하는 월동기(12–2월) 평균기온을 비교한 결과, 2024년 약충의 월동기간인 2023년 12월–2024년 2월의 평균기온은 2.5℃로, 2023년 약충의 월동기간(2022년 12월–2023년 2월, 0.1℃)과 2025년 약충의 월동기간(2024년 12월–2025년 2월, 0.2℃)보다 약 2℃ 이상 높았다. 이러한 상대적으로 온화한 월동기 기온은 2024년 약충의 월동 생존과 봄철 활동에 일부 영향을 미쳤을 가능성이 있다. 다만, 2024년 약충 개체수의 증가는 월동기 기온뿐만 아니라 전년도 유충 발생 규모와 기후ㆍ환경 조건, 겨울철 월동 생존율 등의 이월효과에도 영향을 받았을 것으로 추정하였다. 이번 조사에서도 가장 높은 발생 밀도를 나타낸 2024년에 약충과 유충 개체수가 모두 가장 높게 확인되어 봄철 약충 발생 규모가 해당 연도의 전체 참진드기 밀도 증가와 관련있을 가능성을 시사하였다. 반면, 2024년 유충 발생 규모가 가장 높았음에도 2025년 약충과 전체 참진드기 채집 개체수는 2024년보다 감소하였다. 이는 다음 해 약충의 채집 개체수가 전년도 유충 발생 규모만으로 결정되지 않으며 2025년의 상대적으로 낮은 월동기 및 봄철 기온 등 환경조건이 월동 약충의 생존과 활동에 일부 영향을 미쳤을 가능성을 시사한다. 그러나 유충의 흡혈과 탈피, 월동 생존율 및 기타 환경요인을 직접 조사하거나 통계적으로 분석하지 않았으므로 2025년의 감소 원인을 특정 요인으로 단정하기는 어렵다. 따라서 연도별 참진드기 발생 변동은 당해 연도의 기온뿐만 아니라 전년도 개체군 규모, 월동기 환경 조건, 강수량, 습도, 숙주동물 밀도 및 서식환경 등을 함께 고려하여 신중하게 해석할 필요가 있다[8,12]. 향후 이러한 요인과 참진드기 개체군 변동의 관계를 규명하기 위한 장기적인 연구가 필요하다.

조사기간 동안 월별 발생 양상을 보면, 참진드기 지수는 4–5월에 증가한 이후 6–7월 감소하였다가 8–9월에 다시 급격히 증가하는 공통적인 경향을 나타내었다. 특히 평균기온이 상승하는 시기에 참진드기 지수가 함께 증가하는 경향이 확인되었으며 평균기온이 20℃ 이상으로 유지되는 여름철에 개체수가 크게 증가하였다. 반면 평균기온이 높은 시기임에도 불구하고 6–7월에는 참진드기 지수가 일시적으로 감소하였는데 이는 장마 시기 강수 증가에 따른 서식지 내 환경 변화 등이 참진드기의 활동성에 영향을 미친 것으로 추정된다. 성장단계별로는 약충이 5월, 성충이 7월, 유충이 8–9월에 가장 높은 밀도를 보여 뚜렷한 계절적 차이가 확인되었다. 참진드기는 주로 약충 단계로 월동하므로 봄철은 대부분 약충의 개체수 증가가 관찰되며 6월과 7월에 걸쳐 성충으로 성장하고 산란하고, 8월부터 9월에 산란한 알에서 유충이 부화하면서 개체군의 밀도가 크게 증가하는 것으로 알려져 있다[7]. 특히 국내 참진드기 개체군의 계절적 증가는 유충 발생에 의해 주도되는 것으로 판단된다.

환경별 채집 결과에서는 초지 환경이 모든 연도에서 가장 높은 밀도를 나타냈으며 2023년 20,260개체, 2024년 29,118개체, 2025년 14,595개체가 채집되어 전체 채집 개체수 중 가장 높은 비율을 차지하였다. 이는 초지 환경이 참진드기의 서식과 숙주동물 활동에 적합한 조건을 제공하기 때문으로 생각되며, 이전 감시 결과와도 일치한다[13,14]. 종별로는 2023년 3속 5종, 2024–2025년 3속 6종이 확인되었다. 작은소피참진드기와 개피참진드기는 전국적으로 분포하였으며, 뭉뚝참진드기는 진주시와 울산광역시 등 주로 남부 지역에서 확인되었다. 일본참진드기는 일부 지역을 제외하고는 채집 개체수가 매우 적었으며 사슴피참진드기, 제주피참진드기 및 산림참진드기는 산간 지역 또는 남부 지역과 같은 특정 지역에서 주로 확인되었다[13,14]. 특히 SFTS의 주요 매개종으로 알려진 작은소피참진드기는 국내 우점종임을 다시 한번 확인하였으며, SFTSV 검출이 보고된 개피참진드기, 뭉뚝참진드기 및 일본참진드기 역시 모든 조사 연도에서 확인되었다.

이러한 계절적ㆍ환경별 발생 양상은 이전 국내 감시 결과와도 유사하였다. 2022–2024년 감시자료에서도 약충은 주로 봄철, 성충은 여름철, 유충은 8–9월에 높은 밀도를 보였으며 초지에서 많은 개체가 채집되고 작은소피참진드기가 우점하는 양상이 반복적으로 확인되었다[13-15]. 이는 국내 참진드기의 발육단계별 계절성과 주요 서식환경 및 우점종의 특성이 연도별로 대체로 유지되고 있음을 보여준다. 다만 기존 연도별 보고자료와 이번 연구는 조사 지역 및 집계 범위에 차이가 있으므로 총 채집 개체수와 참진드기 지수의 직접적인 수치 비교에는 주의가 필요하다.

SFTS는 국내에서 매년 지속적으로 환자가 발생하는 대표적인 진드기 매개감염병이다. 국내 선행 감시자료에서는 참진드기 유충의 밀도가 8–9월에 크게 증가한 이후 SFTS 환자 발생이 가을철에 증가하여 10월에 가장 많은 것으로 보고되었다[13-15]. 이번 조사에서도 참진드기 유충의 밀도는 8–9월에 크게 증가하여 선행 감시자료와 유사한 발생 양상을 보였다. 이 시기는 성묘와 농작업 등 야외활동이 증가하는 시기와 겹쳐 사람과 참진드기의 접촉 가능성이 높아질 수 있다. SFTSV는 작은소피참진드기에서 경란형 전파가 가능한 것으로 보고되어[1,16], 유충도 잠재적으로 SFTSV를 보유할 수 있다. 최근 기후변화에 따른 평균기온 상승과 생태환경 변화로 참진드기의 서식 범위와 활동 기간이 확대될 가능성이 제기되고 있으며, 이에 따라 진드기 매개감염병의 발생 위험도 증가할 수 있다[8,9,12]. 이번 연구는 전국 16개 지역에서 수행된 3년간의 감시자료를 통해 국내 참진드기의 연도별ㆍ계절별 발생 특성과 종 분포를 확인하였으며 봄철 약충 발생 규모가 해당 연도의 전체 참진드기 밀도와 관련될 가능성을 제시하였다.

이번 연구는 연도 간 비교를 위해 3개 연도에 공통으로 포함된 16개 지역의 월 1회 감시자료를 분석하였으므로 연도별 전체 조사 지역의 공간적 변이와 단기간의 참진드기 밀도 변화를 충분히 반영하지 못했을 수 있다. 또한 기상ㆍ환경요인과 참진드기 발생 양상을 기술적으로 비교하였고 병원체 검출 결과 및 환자 발생자료와의 연계 분석을 수행하지 않아, 참진드기 밀도 변화의 원인과 감염 위험 간의 직접적인 관련성을 평가하는 데 한계가 있다.

이러한 제한점에도 불구하고 이번 연구는 동일한 16개 지역에서 3년간 수행된 감시자료를 바탕으로 국내 참진드기의 연도별ㆍ계절별 발생 특성과 종 분포를 확인하였다는 데 의의가 있다. 이번 연구 결과는 국내 참진드기 발생 양상을 이해하고 진드기 매개감염병 감시 및 예방 전략을 수립하기 위한 기초자료로 활용될 수 있다. 향후 지속적인 감시와 함께 기후요인, 숙주동물 밀도, 병원체 검출 결과 및 환자 발생자료를 연계한 장기적인 생태학적 연구가 필요하다.

## Figures and Tables

**그림 1:** 2023–2025년 연도별 국내 참진드기의 성장단계에 따른 월별 발생 양상

**그림 2:** 2023–2025년 국내 참진드기 밀도와 기온의 월별 변화

**그림 3:** 2023–2025년 연도별 국내 참진드기 발생 분포 비교

**표 1:** 조사기간(4–11월) 발생단계별 참진드기 채집 개체수(2023–2025년)

연도	발생단계	월별								합계
		4월	5월	6월	7월	8월	9월	10월	11월	
2023	성충	555	706	1,746	4,139	1,122	238	147	71	8,724
	약충	4,171	4,384	2,815	1,157	988	788	118	61	14,482
	유충	33	241	202	307	8,369	9,834	2,584	295	21,865
	소계	4,759	5,331	4,763	5,603	10,479	10,860	2,849	427	45,071
2024	성충	956	1,856	2,141	2,825	1,108	204	240	47	9,377
	약충	6,897	8,860	7,059	1,269	1,535	1,565	150	65	27,400
	유충	137	558	546	1,088	14,594	14,583	3,110	158	34,774
	소계	7,990	11,274	9,746	5,182	17,237	16,352	3,500	270	71,551
2025	성충	320	692	1,315	1,958	809	117	175	73	5,459
	약충	2,869	4,602	2,794	1,345	421	519	245	150	12,945
	유충	29	276	282	1,635	10,967	9,659	3,013	431	26,292
	소계	3,218	5,570	4,391	4,938	12,197	10,295	3,433	654	44,696

**표 2:** 채집환경에 따른 종별 참진드기 채집 개체수(2023–2025년)

연도	종별	환경별				합계
		초지	산길	잡목림	무덤	
2023	작은소피참진드기	10,169	3,090	4,374	4,456	22,089
	개피참진드기	344	276	172	212	1,004
	뭉뚝참진드기	24	14	26	4	68
	일본참진드기	15	5	5	11	36
	사슴피참진드기		1	5	3	9
	유충(피참진드기속)	9,708	3,917	2,105	6,134	21,864
	유충(참진드기속)		1			1
	소계	20,260	7,304	6,687	10,820	45,071
2024	작은소피참진드기	18,745	4,446	7,470	5,096	35,757
	개피참진드기	183	264	167	308	922
	뭉뚝참진드기	22	11	15	13	61
	일본참진드기	10	5	4	6	25
	사슴피참진드기	3	4	1	2	10
	제주피참진드기				2	2
	유충(피참진드기속)	10,155	6,059	5,969	12,590	34,773
	유충(뭉뚝참진드기속)			1		1
	소계	29,118	10,789	13,627	18,017	71,551
2025	작은소피참진드기	7,721	3,052	3,517	3,267	17,557
	개피참진드기	198	271	141	175	785
	뭉뚝참진드기	3	2	6	3	14
	일본참진드기	10	3	5	22	40
	사슴피참진드기	5		1		6
	산림참진드기	2				2
	유충(피참진드기속)	6,656	4,663	6,424	8,549	26,292
	소계	14,595	7,991	10,094	12,016	44,696
